# Full-Field 3D Displacement Measurement of Suspended Ceiling Systems Under Seismic Loading Using a Consumer-Grade Multi-Camera Framework

**DOI:** 10.3390/s26134011

**Published:** 2026-06-24

**Authors:** Mearge Kahsay Seyfu, Yuan-Sen Yang, Cameron C. W. Flude, David T. Lau, Jeffrey Erochko, Hung-Wei Liu

**Affiliations:** 1 Department of Civil Engineering, National Taipei University of Technology, No. 1, Sec. 3, Zhongxiao E. Rd., Daan Dist., Taipei 10608, Taiwan; t112429402@ntut.edu.tw (M.K.S.);; 2 Department of Civil and Environmental Engineering, Carleton University, Ottawa, ON K1S 5B6, Canada; cameronflude@cmail.carleton.ca (C.C.W.F.); davidlau@cunet.carleton.ca (D.T.L.); jeffrey.erochko@carleton.ca (J.E.)

**Keywords:** suspended ceiling, stereo vision, shaking table testing, 3D displacement, measurement accuracy

## Abstract

Suspended ceiling systems are among the most seismically vulnerable non-structural components in buildings, posing significant life-safety risks and economic losses, yet understanding their full-field kinematic behavior under seismic loading remains a major experimental challenge. Conventional contact sensors offer limited spatial coverage and can alter the dynamic properties of lightweight panels due to mass loading. In contrast, non-contact optical alternatives are rarely feasible in shake-table environments due to restricted viewing angles, extensive areal coverage requirements, and the risk of equipment damage from falling panels. This study proposes an end-to-end three-dimensional displacement measurement framework for large-scale shake-table testing of suspended ceiling systems, employing consumer-grade cameras with purpose-built tools that cover the complete experimental workflow, including motion-based video trimming, semi-automated calibration, a robust multi-stage image-tracking pipeline that maintains trajectory continuity under extreme inter-frame displacements, and a ceiling system motion visualization and analysis tool. The framework was validated through a full-scale shake-table experiment continuously tracking 324 spatial nodes across 81 ceiling panels, achieving an RMSE below 3 mm in all spatial directions and exact peak-frequency agreement in 9 out of 10 test cases. A parallel processing architecture reduced total processing time from over 27 h to under 10 min without GPU acceleration, and six-degree-of-freedom rigid-body analysis resolved the complete panel failure sequence from constrained oscillation through multi-axis rotation to gravitational free fall, a level of kinematic detail unattainable with conventional instrumentation. This framework establishes a practical, scalable foundation for full-field seismic performance assessment of non-structural systems where conventional instrumentation is physically or logistically infeasible.

## 1. Introduction

Suspended ceiling systems are among the most common non-structural components in modern buildings, serving functions that include acoustic control, fire resistance, concealment of building services, and interior finishing [1,2]. Despite their non-structural classification, they are highly vulnerable to earthquakes [3,4,5,6]. Post-earthquake reconnaissance studies consistently identify them as the major contributors to building functional failure, occupant risk, and repair costs that frequently exceed those of the structural systems [7,8,9,10]. These failures arise from complex interactions between seismic loading, progressive degradation at grid connections, and the limited lateral and rotational stiffness of conventional lay-in ceiling configurations [9,11]. Understanding and quantifying these failure mechanisms is therefore essential to improving non-structural seismic design provisions and life-safety performance.

Large-scale shake-table testing is the standard experimental approach for evaluating ceiling seismic performance [12,13,14]. It allows full-scale assemblies to be subjected to controlled, repeatable ground motions and enables researchers to observe the progress of damage, from elastic deformation to grid yielding, runner dislodgement, and panel collapse, across lay-in, continuous, and multi-elevation configurations [1,4,15]. The central instrumentation challenge is capturing the spatial and temporal response of the entire ceiling, not just isolated points [14,16]. Conventional contact sensors, LVDTs, wire potentiometers, and accelerometers are accurate but have significant limitations for this application [17]. Their mass alters the dynamic behavior of the lightweight panels they measure, their cabling is difficult to route overhead, and deploying them at sufficient density across a large specimen is practically not feasible [18].

Vision-based methods offer a non-contact measurement alternative [19,20,21]. Digital Image Correlation (DIC) and optical target tracking reconstruct displacement fields by tracking surface features or markers across image sequences without contacting the specimen [22,23]. These methods have been applied successfully to beam deflections [24], inter-story drift [25], multi-story building displacement [26,27], wall shear deformations [22], and bridge pier cracking [28,29,30]. Stereo-vision systems extend this to three-dimensional tracking via geometric triangulation from two synchronized cameras [31,32]. Recent technological improvements in affordable consumer cameras have made multi-camera deployments practical at full experimental scale [33,34]. Despite this track record, application to suspended ceilings remains limited. Guo et al. [35], Wang et al. [36], and Han et al. [37] use point cloud or semantic segmentation approaches to classify the ceiling condition as damaged or undamaged, rather than measuring continuous displacement fields.

Furthermore, these methods are computationally costly, which limits their utility for time-series dynamic monitoring. While conventional DIC implementations are accurate and capable of continuous displacement tracking [19], they require a speckle-coated surface [28], uniform illumination [24], relatively small out-of-plane displacement, a near-perpendicular angle of sight [24], and high-quality images [21]. However, these optical and geometric prerequisites are rarely feasible within the physical constraints of large-scale ceiling shake-table experiments. These gaps arise from geometric, algorithmic, and computational challenges specific to ceiling testing environments: cameras must be positioned within potential debris zones with an oblique line of sight, inter-frame displacements during seismic events are large and rapid, lighting conditions are poor, and multi-camera systems generate substantial data volume.

The primary challenge is algorithmic. The Enhanced Correlation Coefficient (ECC) algorithm [38] is a tool for aligning two images by finding the warp matrix that best matches them. It initializes each frame’s estimate from the previous frame to align, a strategy that works well for slow deformations but fails to converge when inter-frame displacements are large, placing the optimizer outside the basin of convergence of the true solution [39]. This failure mode is particularly critical in seismic testing, where large-amplitude, high-velocity motion is of primary interest. To address this, the present study proposes a multi-step warp initialization strategy based on kinematic extrapolation of the recent displacement history, combined with a cascaded fallback mechanism that is activated when convergence fails. This extends reliable tracking through the pre-failure and progressive connection-failure phases of panel behavior. However, tracking is ultimately lost when panel detachment induces large out-of-plane rotations, a limitation explicitly characterized in the results.

This paper presents an end-to-end stereo-vision framework for full-field three-dimensional kinematic monitoring of suspended ceiling systems under seismic loading. The framework combines consumer-grade cameras with purpose-built algorithms covering the complete workflow: video trimming, calibration, fiducial marker-based correspondence, multi-stage ECC tracking, stereo synchronization, triangulation, and six-degree-of-freedom (6-DOF) motion extraction. The three primary contributions are: (1) the multi-step warp initialization and cascaded fallback pipeline, which extends reliable tracking well beyond conventional ECC and standard DIC implementations up to the limits of the planar template assumption; (2) a parallel processing architecture that reduces processing time from over a day to a few minutes without GPU acceleration, making dense stereo tracking practical within standard post-processing workflows; and (3) a comprehensive experimental demonstration of full-field three-dimensional kinematic monitoring of a complete suspended ceiling system, tracking hundreds of nodes across multiple panels through progressive failure and collapse, validated against independent accelerometer measurements with acceptable accuracy across all spatial axes.

The rest of this paper is organized as follows. Section 2 details the complete methodological framework. Section 3 presents the experimental validation results and ceiling system displacement analysis. Section 4 provides concluding remarks, limitations, and directions for future research.

## 2. Materials and Methods

**Figure 1:**
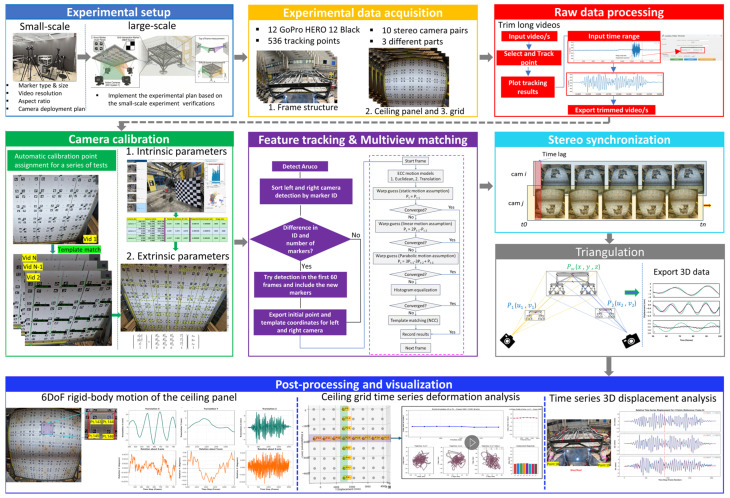
Methodological framework.

The proposed vision-based full-field displacement and rigid body kinematics measurement system for suspended ceilings follows a comprehensive multi-stage methodological workflow, as illustrated in Figure 1. The pipeline encompasses eight sequential stages: (1) experimental setup, (2) experimental data acquisition, (3) raw data processing, (4) camera calibration, (5) feature tracking and multiview matching, (6) stereo synchronization, (7) triangulation, and (8) post-processing and visualization. Each stage is described in detail in the following subsections.

### 2.1. Experimental Setup

#### 2.1.1. Small-Scale Shake-Table Experiment

**Figure 2:**
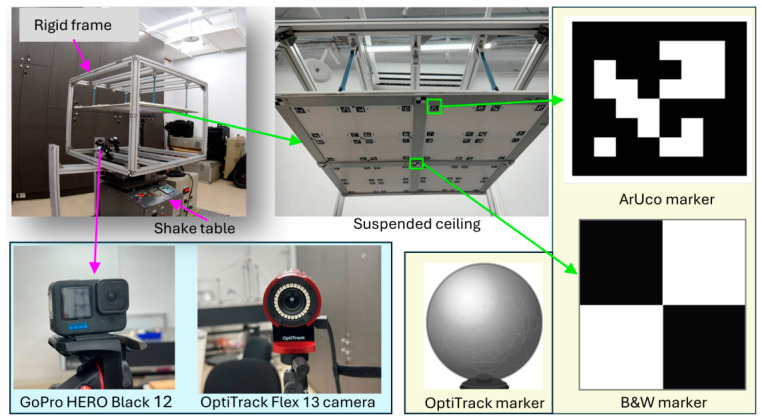
Experimental model, cameras, and markers used in the small-scale shake-table experiment.

Large-scale shake-table experiments are resource-intensive and costly to repeat, so deficiencies in instrumentation identified after testing cannot be corrected retrospectively. A preliminary experiment on a scaled-down ceiling specimen was therefore conducted to resolve practical design decisions specific to ceiling-system measurement and to validate the image-based tracking pipeline before full-scale deployment. The experimental model is a geometrically scaled replica of the suspended-ceiling testing platform at Carleton University’s Multi-Hazard Research Center; further details are available in Flude et al. [40]. The supporting frame measured 70 cm in width, 70 cm in length, and 50 cm in height, representing an approximate 1:8 scale of the full-scale structure, and was assembled from 30 mm × 30 mm aluminum extruded profiles. A 65 cm × 65 cm ceiling model was suspended beneath the frame via five non-rigid 7 cm long connectors, as shown in Figure 2, thereby introducing lateral flexibility for dynamic displacement monitoring.

Capturing the full extent of a suspended-ceiling area with below-mounted cameras is a challenging geometric problem due to the test facility’s confined clearances. Camera standoff distances and viewing angles were determined through systematic empirical and analytical evaluation guided by three monitoring objectives: side-mounted cameras observing the lateral faces of the rigid frame, top-mounted cameras covering the upper chords, and upward-facing cameras providing full plan coverage of the ceiling surface. Each component presented distinct geometric and photometric conditions, so markers were assigned independently based on camera working distance, angle, available field of view, and the specimen’s physical appearance. Black-and-white square targets were used on the ceiling grid; ArUco fiducial markers [41] were used on the ceiling panels and rigid frame. Marker dimensions were iteratively tested to determine the minimum size sustaining reliable detection under experimental conditions. The imaging setup comprised three GoPro HERO 12 Black cameras (GoPro, Inc., San Mateo, CA, USA) mounted beneath the ceiling, facing upward: two targeting the top of the rigid frame and two targeting its sides. Three-dimensional displacements computed by the framework were validated against OptiTrack motion-capture (NaturalPoint Inc, Corvallis, OR, USA) measurements.

#### 2.1.2. Large-Scale Shake-Table Experiment

A series of experiments was conducted at Carleton University’s seismic laboratory utilizing a shaking table system containing four independent motion platforms capable of simulating six degrees of freedom. The platforms are mechanically connected to a rigid latticed steel frame measuring 5.4 m × 5.4 m in plan and 1.735 m in height, designed to replicate the structural bay of a typical building floor [40]. The rigid frame’s fundamental frequency exceeds 20 Hz, ensuring that seismic input signals are transmitted to the specimen with minimal distortion. The four platforms operate in synchronization to drive the entire frame assembly; the present study employs three-directional synchronized motion to represent realistic seismic excitation.

**Figure 3:**
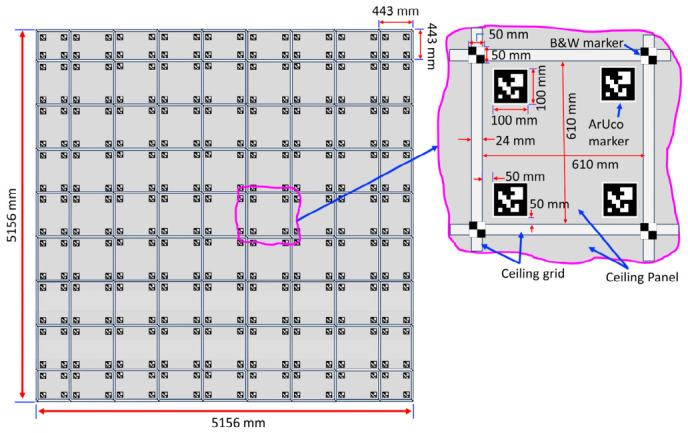
Ceiling configuration and markers used in the study.

**Figure 4:**
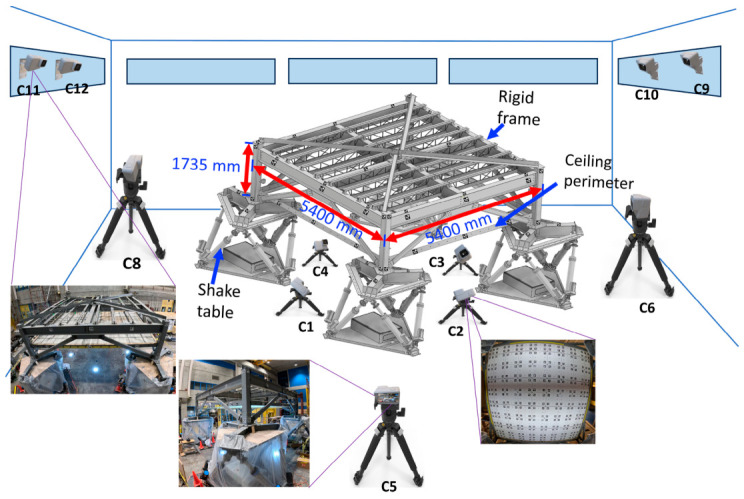
Experimental model and camera arrangement used in the large-scale shake-table experiment.

The suspended ceiling specimen is installed beneath the steel grid frame, with a clearance of 2.10 m above ground. The ceiling occupies a planar area of 5.156 m × 5.156 m and consists of 81 panels arranged in a 9 × 9 layout. Interior panels measure 610 mm × 610 mm, while perimeter panels have a reduced width of 443 mm (Figure 3). The ceiling grid used is a light-gauge steel with a nominal width of 24 mm. DICT_6 × 6_1000 ArUco markers with a dimension of 100 mm × 100 mm are used as tracking targets on the ceiling panel. The markers were stuck at each corner of every ceiling panel, as illustrated in Figure 3; the same marker type is used on the rigid frame. Square black-and-white markers, each 50 mm × 50 mm, are affixed at each intersection of the ceiling grids, as shown in Figure 3. The marker size, type, and placement were determined based on the results of the small-scale experiment to ensure reliable detection across the full range of seismic excitation intensities. Three camera deployment configurations were used to capture the three instrumented components of the shaking table systems as shown in Figure 4: four cameras mounted beneath the ceiling facing upward to capture ceiling panel motion; four cameras positioned at the sides of the rigid frame, each capturing two faces of the structure to form a stereo pair; and two stereo pairs mounted at opposite ends of the building’s second floor to capture the top and sides of the rigid frame and the shake-table feet.

### 2.2. Experimental Data Acquisition

Twelve GoPro HERO12 Black cameras were used to record experimental videos, as shown in Figure 4. Based on the analytical and empirical evaluation of the small-scale experiment, the following optimal camera settings are used: 4K resolution with a wide-angle lens, an 8:7 aspect ratio (3840 × 3360 pixels), and a 60 Hz sampling rate.

Consumer-grade cameras do not support hardware trigger mechanisms, so each camera was manually powered on and off independently. This caused each camera to record continuously for 5 to 15 min, even though individual shake events lasted 30 to 45 s. As a result, 684 video files and 57 test runs across 12 cameras were retrieved, amounting to more than 1.7 terabytes of raw data. Using these raw videos directly for image analysis is computationally prohibitive, necessitating the trimming procedure described in the following section. 

### 2.3. Raw Data Processing

Processing this volume of data required careful inspection to identify camera view interruptions, unintended shutdowns, and gaps in image coverage; videos with these problems were excluded from analysis. The remaining videos were trimmed to retain only the frames that span the relevant motion event, using version 1.0.0 of a purpose-built Python-based trimming tool that supports two approaches to identifying trim boundaries. In the first, the operator visually inspected the video and manually entered start and end points in seconds or frame numbers. In the second, the operator selected a region of interest in the first frame and tracked it across all frames using either ECC tracking or ArUco marker detection; the resulting X- and Y-displacement trajectories were plotted frame-by-frame, allowing the operator to identify motion onset and end directly from the displacement signal. Once boundaries were determined, videos were trimmed using FFmpeg (version 8.0.1) without re-encoding, preserving the original image quality. Batch processing was implemented by running each video’s tracking computation in a separate process using Python’s multiprocessing module. After trimming, the 1.7 TB of raw data was reduced to 235 GB, approximately 14% of the original volume, thereby substantially reducing storage requirements and downstream computational costs.

### 2.4. Camera Calibration

Accurate camera calibration is essential for reliable three-dimensional displacement reconstruction, as it establishes the geometric mapping between the physical object location and the image measurement [22,42]. In this study, following image data acquisition, a rigorous camera calibration procedure was performed to enable accurate three-dimensional estimation of structural displacements. The calibration process consisted of two sequential stages: (i) estimation of intrinsic camera parameters using a planar checkerboard target, and (ii) estimation of the extrinsic parameters using known three-dimensional control points within the measurement domain.

The intrinsic parameters of each camera were first determined independently using 25 to 30 images of a planar checkerboard pattern captured at different orientations and positions within the field of view, a standard approach chosen for its practical efficiency compared to complex 3D calibration rigs [43]. This step provides the camera matrix together with the lens distortion coefficients. These parameters describe the internal geometry of the camera’s imaging system, including focal length, principal point, and optical distortions, and are assumed to remain constant for a fixed camera configuration [44].

Following intrinsic calibration, the extrinsic parameters (rotation matrix and translation vector) were estimated to map the camera’s position and orientation relative to the global coordinate system, using a set of control points with known world coordinates. The accuracy of the estimated parameters was evaluated using the mean reprojection error, defined as the Euclidean distance between observed and reprojected image points. This is important to ensure the calibration results meet the required precision. The complete set of geometric parameters was optimized by minimizing the mean reprojection error using a nonlinear least-squares Perspective-n-Point (PnP) framework based on the standard pinhole camera model [45]. This two-stage approach ensures that both the camera’s internal imaging characteristics and its spatial relationship to the global coordinate system are accurately defined, which is essential for reliable triangulation and displacement measurement [22]. The camera calibration process yielded an average reprojection mean error of 2 to 3 pixels across the experimental test cases, representing 3 to 5 mm in the physical domain. It is important to note that while the use of consumer-grade, ultra-wide-angle lenses inherently introduces this baseline uncertainty in absolute spatial positioning, the proposed framework is designed to capture relative dynamic displacements. Because the kinematic tracking calculates differential, frame-to-frame movement, this static absolute calibration offset does not compromise the sub-millimeter accuracy of the transient structural measurements.

#### Automated Calibration Point Propagation Between Test Sessions

**Figure 5:**
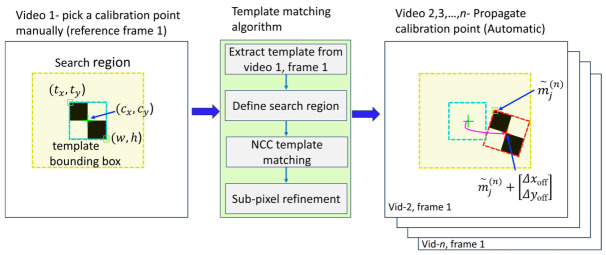
Automated control point transfer from the reference video to successive videos in a test session.

During sequential shake-table experiments, small positional disturbances to consumer-grade cameras caused by power cycling or test setup adjustments can invalidate the direct reuse of extrinsic calibration coordinates across different test sessions. Because manual re-annotation for every video is prohibitively time-consuming, an automated calibration point propagation algorithm was developed to transfer control-point coordinates from a reference session to all subsequent sessions, using subpixel Normalized Cross Correlation (NCC) template matching [39], as depicted in Figure 5.

For each of the m calibration control points, the operator specifies the sub-pixel calibration center $\left(c_{x},c_{y}\right)$ and defines an associated rectangular template region with the top-left coordinate $\left(t_{x},t_{y}\right)$ and dimensions w×h on the reference video. The fixed geometric offset between the bounding box origin and the calibration center is given by $\Delta x_{\mathrm{off}}=c_{x}-t_{x},\;\Delta y_{\mathrm{off}}=c_{y}-t_{y}$. For each subsequent video n, a template patch is extracted from the reference frame and matched within a search region using NCC template match [39]. Then, the optimal match $\left(x^{*},y^{*}\right)$ is refined to sub-pixel precision using parabolic int erpolation as defined by Equation (1).

(1) $$\delta x=\frac{R(x^{*}+1,y^{*})-R(x^{*}-1,y^{*})}{2[2R(x^{*},y^{*})-R(x^{*}+1,y^{*})-R(x^{*}-1,y^{*})]}\;,$$

and similarly, for δy, yielding the refined position. Here, $\left(x^{*},y^{*}\right)$ represents the integer coordinate yielding the maximum NCC score, and $R(x^{*},y^{*})$ is the corresponding peak value. By evaluating the adjacent correlation scores along each axis, the sub-pixel offsets δx and δy are isolated, yielding the refined template coordinate ${\overset{\sim }{m}}_{j}^{\left(n\right)}=(x^{*}+\delta x,\;{\;y}^{*}+\delta y)$ for the j-th control point in the n-th frame. The propagated calibration center ($p_{cal,j}^{\left(n\right)}$) is then obtained using Equation (2).

(2) $$p_{\mathrm{cal},j}^{\left(n\right)}={\overset{\sim }{m}}_{j}^{\left(n\right)}+\left[\begin{array}{c} \Delta x_{\mathrm{off}}\\ \Delta y_{\mathrm{off}} \end{array}\right],$$

To mitigate the influence of occlusions and false matches, an outlier rejection framework is applied to the calculated Euclidean displacement, $d_{i}={∥p_{\mathrm{cal},i}^{\left(n\right)}-p_{\mathrm{ref},i}∥}_{2}$, where $p_{ref,i}$ denotes the static reference coordinate. A control point is classified as an outlier if its photometric match score falls below the predefined reliability threshold $\rho_{i}<0.6$, or if its spatial displacement exceeds the robust statistical bound defined by the median absolute deviation: $d_{i}>\mathrm{med}(d)+3\cdot \mathrm{MAD}\left(d\right)$, where $\mathrm{MAD}(d)={\mathrm{med}}_{i}(\text{∣}d_{i}-\mathrm{med}(d)\text{∣})$ is the median absolute deviation. This criterion provides robustness against isolated matching failures and avoids sensitivity to outlier-contaminated mean statistics. Points flagged as outliers are subjected to manual verification before acceptance. All accepted calibration sets are further assessed for accuracy using reprojection error. This automated propagation framework reduces calibration effort from a labor-intensive manual process to an efficient automated workflow, making it particularly suitable for large-scale experimental campaigns involving repeated measurements.

### 2.5. Feature Tracking and Multiview Matching

To extract the high-fidelity displacement response of the suspended ceiling system, a customized, multi-stage image tracking pipeline was developed. This architecture integrates automated stereoscopic initialization, gradient-based optimization, and structural kinematics to maintain robust trajectory data during severe seismic events. All image processing was performed on a laptop workstation equipped with an Intel Core i7-11800H processor (8 cores, 16 threads, 2.3 GHz base clock), 64 GB DDR4-3200 RAM, running Windows 11. No GPU acceleration was employed; all computations were executed on the CPU using Python and OpenCV’s (version 4.12.0) multi-threaded processing pipeline.

#### 2.5.1. Multiview Matching

Before tracking, an automated initialization protocol detects ArUco markers across the stereo camera array to extract initial spatial coordinates and define reference templates. To ensure reliable detection under uneven lighting and sensor noise, frames are preprocessed through Contrast Limited Adaptive Histogram Equalization (CLAHE), non-local mean denoising, and high-pass sharpening. If markers remain undetected, the system applies secondary thresholding methods, Otsu’s method [46] and Adaptive Gaussian thresholding, in sequence. Upon successful detection, sub-pixel corner refinement computes the centroid of each marker, and a reference template is extracted using a 5-pixel padded bounding box around these coordinates. To handle initial occlusions, the algorithm prioritizes the first frame while scanning frames 2 through 60 to recover missing targets. The dataset is then filtered to retain only markers detected in both left and right camera views, a requirement for stereo triangulation. Final template coordinates are exported as CSV files for use in the tracking stage.

#### 2.5.2. Image Tracking

Tracking markers across thousands of frames under extreme seismic motion requires a tracking mechanism that is both computationally efficient and robust to large inter-frame displacements. The ECC algorithm [38,39] aligns a predefined reference template with successive video frames by maximizing the zero-mean normalized cross-correlation. For a given reference pixel coordinate vector $x_{r}=[x_{r},y_{r}{]}^{T}$, the algorithm iteratively optimizes a parameter vector p to compute a parametric warp matrix W(p) that minimizes the photometric discrepancy between the target and the reference images. To account for the dynamic behavior of the ceiling panels, the proposed tracking system primarily uses a Euclidean motion model that allows 2D translation and in-plane rotation, as defined in Equation (3).

(3) $$\left[\begin{array}{c} x_{t}\\ y_{t} \end{array}\right]=W(p)x_{r}\Leftrightarrow \left[\begin{array}{ccc} cos(\theta ) & -sin(\theta ) & t_{x}\\ sin(\theta ) & cos(\theta ) & t_{y} \end{array}\right]\left[\begin{array}{c} \begin{array}{c} x_{r}\\ y_{r} \end{array}\\ 1 \end{array}\right],$$

where $\left(x_{t},y_{t}\right)$ are the warped coordinates in the target image, obtained by applying the warp matrix W(p) to the reference image coordinate $\left(x_{r},y_{r}\right)$. The warp matrix W(p) is parameterized by $p=[\theta ,t_{x},t_{y}{]}^{T}$, where $t_{x}$ and $t_{y}$ represent spatial translations and θ denotes the in-plane rotation. While highly accurate for continuous micro-vibrations, the standard ECC algorithm is computationally expensive and highly susceptible to local minima if subjected to the extreme displacements and high-frequency motion of large-scale shake-table testing.

To resolve this, a three-phase optimization-and-fallback architecture was implemented. First, to eliminate the computational bottleneck of global image searching across thousands of high-resolution images, the algorithm restricts the ECC evaluation to a localized Region of Interest (ROI) cropped with a pixel margin (δ) around the target’s last known spatial coordinate. This localized bounding box ($I_{crop}=I(x\pm \delta ,y\pm \delta$)) significantly reduces the number of pixels evaluated in each frame, serving as the primary driver of the system’s high-speed computational output. The ECC objective function maximizes the correlation coefficient ρ between the zero-mean template $\overset{\_}{T}$ and the warped zero-mean cropped image ${\bar{I}}_{crop}(W\left(p\right)x_{r})$, as denoted by Equation (4).

(4) $${max}_{p}\rho (p)=\frac{\left\langle \overset{\_}{T},{\overset{\_}{I}}_{crop}(W(p)x_{r})\right\rangle }{∥\overset{\_}{T}∥\cdot ∥{\overset{\_}{I}}_{crop}(W(p)x_{r})∥},$$

Second, a kinematic-informed warp initialization mechanism was introduced to prevent the gradient-based optimizer from failing during sudden, high-velocity displacements. Rather than assuming a zero-displacement starting condition, the algorithm leverages structural kinematics to predict the target’s current position based on its immediate temporal history. During continuous tracking, the initial warp guess is informed by a first-order velocity prediction. If the optimizer fails to converge, the system automatically escalates to a second-order polynomial extrapolation to account for structural acceleration, projecting the spatial coordinate in the current frame $x_{i}$ using the target’s trajectory across the three preceding frames ($x_{i-1}{,\;x}_{i-2}{\;and\;x}_{i-3}$), as denoted by Equation (5).

(5) $$x_{i}=3x_{i-1}-3x_{i-2}+x_{i-3},$$

Third, to mitigate tracking failures induced by severe motion blur during peak seismic excitation, the algorithm employs an iterative spatial smoothing protocol. If raw gradients fail to align due to high-frequency noise, the system sequentially applies Gaussian filters to the reference template. While this aggressive smoothing can degrade sub-pixel localization accuracy, it effectively simulates the experimental motion blur to force convergence.

Despite these kinematic and photometric optimizations, continuous tracking can still be abruptly disrupted during localized structural failures. When a ceiling panel physically detaches, it undergoes nonlinear, out-of-plane 3D rotation and substantial scale changes that completely invalidate the 2D planar assumption of the ECC template. To ensure uninterrupted data capture during these critical failure events, an adaptive, multi-stage fallback strategy was integrated into the algorithm. If the ECC correlation coefficient falls below a strict 0.9 confidence threshold, the system first attempts to normalize localized shadow disruptions using histogram equalization, then transitions to NCC template matching for robust 2D translation recovery. The maximum inter-frame displacement that the tracking algorithm can reliably handle is governed by the search region, defined as a margin in each direction from the previously tracked position.

### 2.6. Camera Synchronization

Synchronization is a critical step in image measurement. However, consumer-grade cameras do not support a trigger mechanism for instant stereo recording [47]. To address this, a sub-frame level synchronization method, which was developed by Seyfu and Yang [33], is adopted. Their approach first identifies a frame-level time lag that minimizes the overall triangulation reprojection error. It then achieves sub-frame precision by modeling the localized projection error as a quadratic curve and performing interpolation directly on the 2D image points. By pinpointing the exact minimum of this fitted curve, the algorithm corrects sub-frame timing discrepancies.

Prior experimental validation of this method demonstrated that this sub-frame refinement reduces the Root Mean Square Error (RMSE) of stereo triangulation by up to 79% relative to standard integer-frame alignment, quantitatively bounding the maximum synchronization-induced triangulation or spatial uncertainty to less than 1 mm. Because the peak transient displacements of the suspended ceiling system during seismic excitation significantly exceed this margin, this residual spatial uncertainty is mathematically negligible, and the effect on the interpretation of the dynamic structural response is minimal. Consequently, this methodology allows consumer-grade optical setups to maintain high-fidelity measurement accuracy comparable to expensive, hardware-triggered motion capture systems. For further details on this process, refer to the study conducted by Seyfu and Yang [33].

### 2.7. Stereo Triangulation

Three-dimensional displacement reconstruction was performed by stereo triangulation of the synchronized image-plane coordinate sequences from the left and right cameras. For each Point of Interest (POI) and each frame, the corresponding image coordinates $\left(u_{i},v_{i}\right)$ and $\left(u_{j},v_{j}\right)$ from the left and right views, respectively, were combined with the calibrated camera projection matrices P_1_ and P_2_ to solve for the three-dimensional world coordinates P_u_ (X, Y, Z) using the Direct Linear Transform (DLT) method. The resulting per-frame three-dimensional position estimates were differenced relative to the initial static reference position to yield the absolute three-dimensional displacement time history for each POI. The triangulation procedure was applied independently to all instrumented POIs across the ceiling specimen, yielding spatially distributed three-dimensional displacement fields for each test. The reconstructed displacement time histories capture translational motion in all three spatial directions (X, Y, Z), providing a complete characterization of the dynamic response of the suspended ceiling system under seismic excitation. The triangulated results were exported to CSV files, which serve as input to the post-processing and visualization stage.

### 2.8. Post-Processing and Visualization

To synthesize and interpret the high-volume kinematic datasets generated during image processing, a custom Python-based analytical framework was developed. This interactive spatiotemporal visualization tool bridges the gap between raw optical tracking outputs and meaningful structural interpretations, enabling multidimensional analysis of the suspended ceiling system’s dynamic behavior.

#### 2.8.1. Data Conditioning and Structuring

The post-processing pipeline ingests raw 3D coordinate time-history data discussed in Section 2.7 above. To ensure continuity in the time series displacement visualization, the algorithm automatically detects invalid frames and applies a robust Not a Number (NaN)-handling protocol. This involves bidirectional linear interpolation combined with forward and backward filling, guaranteeing a continuous, unbroken data matrix structured by frame, target ID, and spatial dimensions (X, Y, Z). NaN filling and interpolation were only applied if the consecutive data gap spanned less than 5% of a full vibration cycle. Because this mathematical filling is strictly bound to a negligible fraction of the physical dynamic period, the spectral integrity and peak displacement characteristics of the signals are fully preserved. All subsequent analyses isolate the dynamic response by zeroing the initial coordinate states, converting absolute spatial data into relative time-history displacements. While this adaptive NaN-handling protocol is critical for maintaining data continuity during severe optical disruptions in broader applications, the proposed tracking algorithm achieved 100% temporal continuity in the present study. Consequently, the interpolation protocol was not triggered for the current experimental dataset.

#### 2.8.2. Analytical Visualization Modules

To capture the complex mechanics of the ceiling panels, the interpretation framework is divided into several specialized analytical modules:

- Dynamic Spatial Profiling: The system generates 2D cross-sectional profiles and in-plane transverse plots, allowing for the direct observation of out-of-plane (Z-axis) wave propagation across the ceiling grid along its primary X or Y axes.
- Kinematic Trajectory Tracking: For granular interpretation of individual component behavior, the tool extracts continuous 2D orbital trajectories (Z vs. Y, Z vs. X, and X vs. Y) and magnitude bar charts. This allows precise isolation of highly localized chaotic motions or sudden detachment events in specific panels.
- Time-Series Extraction: The framework generates synchronized, multi-target chronological time-history plots for orthogonal displacements $\left(U_{x},U_{y},U_{z}\right)$

, providing direct comparisons against the input seismic drive signals.

#### 2.8.3. 6-DOF Rigid Body Motion Extraction

Understanding the dynamic response of lay-in ceiling panels under seismic excitation is fundamental to advancing the design and performance assessment of suspended ceiling systems. A critical challenge in evaluating the suspended ceiling is isolating localized lay-in panel motion from the global swinging or twisting of the testing frame. To address this, the framework integrates the Kabsch algorithm [48], which uses Singular Value Decomposition (SVD) to compute the optimal rigid-body transformation matrix between successive frames. By selecting four corresponding point pairs between successive frames with 3-DOF coordinates each, the tool mathematically extracts the 6-DOF rigid body motion, quantifying pure global translations and Euler rotation angles. This isolation allows researchers to accurately decouple the suspension system’s global pendulum motion from the localized, inter-panel relative deformations.

## 3. Results

### 3.1. Tracking Algorithm Validation

The ECC algorithm is an intensity-based image registration method that requires an initial warp estimate, from which it performs an iterative, gradient-based optimization to converge on the true spatial displacement [36]. This process is inherently local; the algorithm finds the correct solution only if the initial guess places the target within a finite spatial neighborhood of its true location, the convergence basin. Under quasi-static or low-velocity conditions, this assumption holds reliably because successive frames differ only by small displacements. During high-intensity seismic excitation, however, inter-frame motion can carry the target entirely outside this basin, causing the optimizer to diverge and tracking to fail.

**Figure 6:**
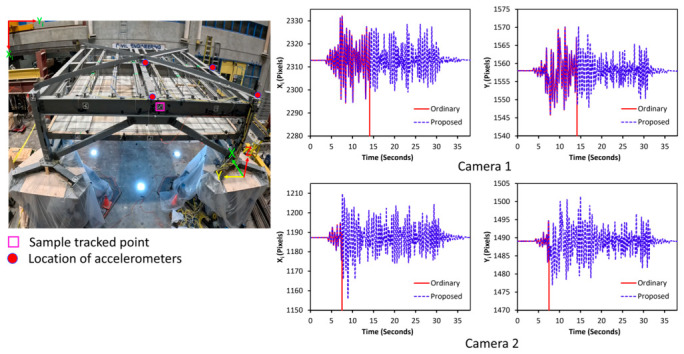
Tracking results comparison between the ordinary and the proposed tracking method for a selected point on the frame structure.

**Figure 7:**
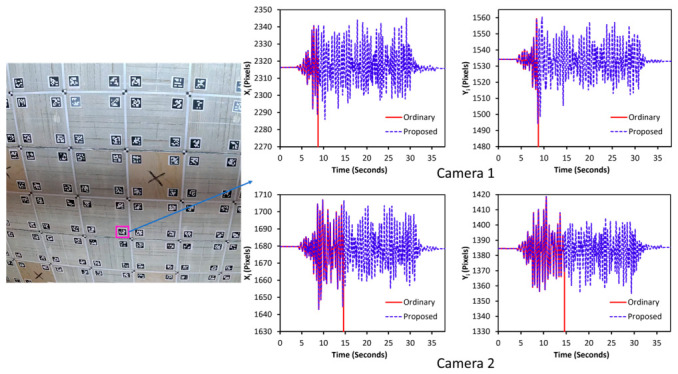
Tracking results comparison between the ordinary and the proposed tracking method for a selected point on the ceiling panel.

Figure 6 and Figure 7 present the comparative displacement time histories of a control point on the rigid frame and the ceiling panel, respectively, explicitly validating the necessity of the proposed framework. As illustrated, the standard ECC approach (red line) experiences catastrophic tracking failures during peak excitation phases when inter-frame displacements exceed the convergence basin, resulting in severe data dropouts. In fact, when using the ordinary tracking method, tracked points experienced severe drift or complete localization failure in 7 out of the 10 test cases. In contrast, the proposed multi-step warp initialization method (blue line) maintains continuous tracking fidelity. By extrapolating the target’s trajectory from multiple preceding frames, the proposed method dynamically pre-aligns the initial warp estimate, ensuring the target remains within the optimizer’s capture range despite sudden structural accelerations. Consequently, the proposed method achieved a 100% tracking success rate across the entire experimental campaign, with zero frames or data points lost to tracking failure. Moreover, about 0.01% of the tracking success was contributed by the NCC fallback, which ensures continuous tracking if ECC optimization fails or yields a correlation below 0.9. This kinematic predictive capability guarantees uninterrupted data acquisition throughout highly nonlinear structural responses, including panel detachment and failure mechanisms, which constitute the most critical events of experimental interest. Furthermore, by dynamically cropping the reference image to a localized margin around the target template, the proposed method reduces the computational cost of tracking by a factor of approximately 89×. A detailed quantitative assessment of this computational efficiency is provided in Section 3.3. 

### 3.2. Experimental Validation

**Figure 8:**
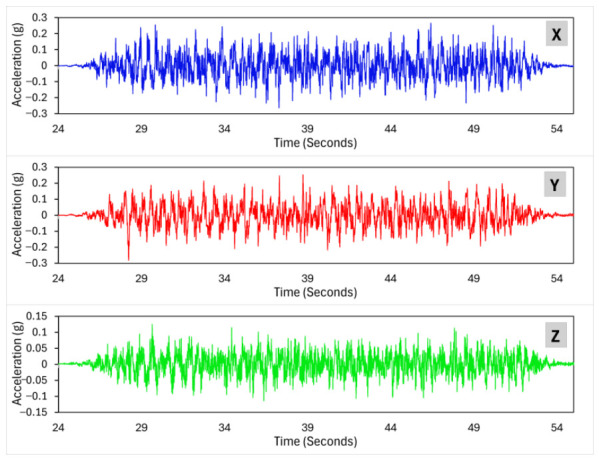
Time-series acceleration data of the triaxial accelerometer.

The displacement responses obtained from the image-based measurement system were compared against reference measurements derived from triaxial accelerometers used in the large-scale experiment. The acceleration signal was corrected for sensor bias by subtracting its mean value before applying ω-arithmetic integration. Then, the accelerometer signals, originally recorded at 1024 Hz, were converted into displacement using the frequency-domain integration (ω-arithmetic) method with a band-pass filter between 0.3 and 40 Hz. While necessary for signal stability, this filtering introduces inherent sensitivity to the selected cutoff frequencies, which can result in localized amplitude distortion, particularly for displacement components near the filter boundaries. The image-based displacement signals, acquired at 60 Hz, were resampled to align temporally with the accelerometer-derived signals, and synchronization was performed using cross-correlation. Figure 8 shows a typical 3D acceleration data from a triaxial accelerometer; this data is typically for the accelerometer located near the sample tracked point depicted in Figure 6.

The performance of the image-based measurement method was quantitatively evaluated against the reference sensor data using several statistical indicators. RMSE is used to quantify the overall magnitude of deviation between the image-based (IM) and accelerometer-based measurement (AM), with N samples, and is defined by Equation (6).

(6) $$\mathrm{RMSE}=\sqrt{\frac{1}{N}\sum_{i=1}^{N}{\left(IM-AM\right)}^{2}},$$

To enable comparison across tests with different displacement amplitudes, the Normalized RMSE (NRMSE) is computed as represented by Equation (7).

(7) $$\mathrm{NRMSE}=\frac{\mathrm{RMSE}}{{AM}_{max}-{AM}_{min}}\times 100\%,$$

The Mean Absolute Error (MAE) reflects the average magnitude of error without heavily penalizing large outliers, and it is represented by Equation (8).

(8) $$\mathrm{MAE}=\frac{1}{N}\sum_{i=1}^{N}|IM-AM|,$$

The peak error is critical for assessing the accuracy of extreme response estimation; it is defined as the absolute difference between the maximum responses and can be calculated by Equation (9).

(9) $$\mathrm{Peak}\;\mathrm{Error}=|\max(x^{IM})-\max(x^{AM})|,$$

In addition, the dominant frequency was extracted from the displacement signals using frequency-domain analysis, and the frequency error was calculated as shown in Equation (10).

(10) $$\mathrm{Frequency}\;\mathrm{Error}=\frac{|f^{IM}-f^{AM}|}{f^{AM}}\times 100\%,$$

where $f^{IM}$ and $f^{AM}$ are the dominant frequencies of the image-based measurement and sensor-based displacement, respectively. A summary of the computed statistical indicators for 10 test cases in the X, Y, and Z directions is presented in Table 1, Table 2 and Table 3, respectively.

#### 3.2.1. Displacement Accuracy

The displacement accuracy of the proposed framework was first assessed in a small-scale preliminary experiment, as illustrated in Figure 2, in which the image-based measurements were validated against OptiTrack motion-capture reference data. The results demonstrated sub-millimeter accuracy across all spatial directions in all test cases, confirming the tracking pipeline’s reliability under controlled conditions before full-scale deployment. The accuracy of the proposed vision-based displacement measurement method was rigorously evaluated by comparing it against the accelerometer baseline. The measurement accuracy of the proposed method for X (out-of-plane), Y (in-plane horizontal), and Z (in-plane vertical) directions is summarized in Table 1, Table 2 and Table 3, respectively. To understand the operational limits and characteristics of the system, the tracking errors were analyzed with respect to three primary variables: the direction of motion, the magnitude of the displacement range, and the dominant structural frequency. The stereo baseline distance for all test cases presented here was 2.32 m.

The proposed method (IM) achieved NRMSE values of 1.2–3.1% in the X-direction, 1.2–2.3% in the Y-direction, and 1.5–5.5% in the Z-direction across all 10 tests when compared with baseline measurement (AM). Although RMSE values increase with displacement range, NRMSE remains broadly stable across configurations, indicating that measurement accuracy scales proportionally with displacement amplitude rather than degrading at larger excitations. The error in the Y-direction is consistently the lowest, with peak values ranging from 0.093 mm (Test 1) to 3.647 mm (Test 7). The X-direction produced the largest errors, reaching 11.216 mm in Test 7, more than three times the corresponding Y-direction peak. This disparity reflects the sensitivity of out-of-plane triangulation at large working distances, where small disparity estimation errors are amplified nonlinearly into depth.

A positive relationship between displacement range and absolute error is observed across all three directions. In the X-direction, RMSE increases from 0.391 mm at a range of 26.760 mm (Test 1) to 2.548 mm at a range of 82.170 mm (Test 7); in the Z-direction, the same progression yields RMSE values from 0.217 mm to 2.970 mm. Displacement amplitudes with larger magnitudes induce larger pixel displacement between successive frames; this phenomenon increases the ECC tracker’s susceptibility to degraded correlation. NRMSE in the Y-direction remains stable across this range (1.22–2.33%), confirming that in-plane tracking accuracy scales well with amplitude. The Z-direction shows greater sensitivity, with NRMSE ranging from 1.47% to 5.50%, reflecting its combined susceptibility to depth reconstruction geometry and large inter-frame displacements.

**Figure 9:**
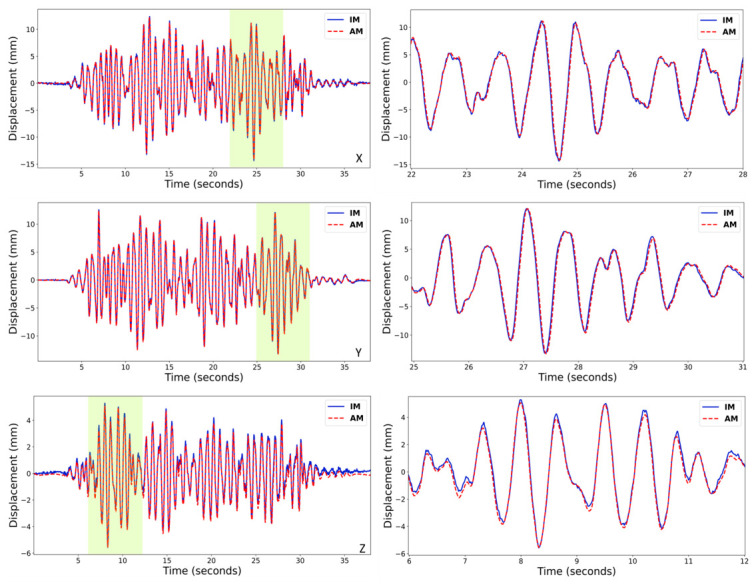
Time-series displacement comparison between AM and IM for the X, Y, and Z axes for Test 1. Full test durations are shown on the **left**, with shaded areas corresponding to the magnified peak responses detailed on the **right**.

**Figure 10:**
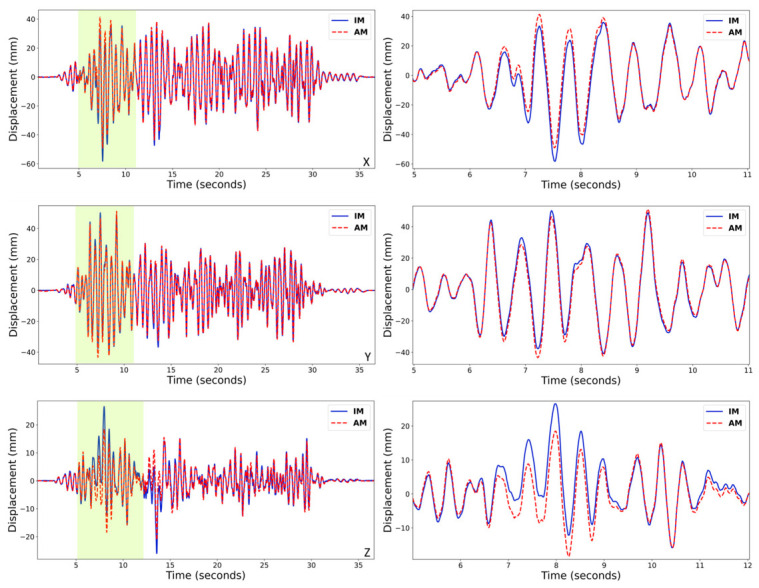
Time-series displacement comparison between AM and IM for the X, Y, and Z axes for Test 10. Full test durations are shown on the **left**, with shaded areas corresponding to the magnified peak responses detailed on the **right**.

Figure 9 and Figure 10 present representative time-series displacement comparisons between the IM and the AM for all three axes, illustrating the best- and worst-performing cases, respectively. In Test 1 (Figure 9), the IM displacement follows the AM reference closely across all three directions throughout the full test duration, with the magnified peak-response windows confirming tight agreement in both amplitude and phase. This demonstrates the framework’s ability to maintain accurate tracking under moderate excitation levels. Test 10 (Figure 10) represents the worst case, with larger displacement amplitudes. In the X- and Y-directions, the IM and AM waveforms remain in close agreement during the peak excitation phase, though minor amplitude discrepancies are visible in the magnified windows. The Z-direction shows the most notable divergence, with a visible drift between the IM and AM signals during the high-amplitude phase, which converges back as the amplitude decreases, consistent with the increased depth reconstruction error observed at higher excitation levels. Overall, the system achieves 2–3% NRMSE in both in-plane and depth directions under most configurations. Residual errors are attributable to depth reconstruction sensitivity at large working distances and inter-frame displacement magnitude at higher excitation levels.

#### 3.2.2. Frequency-Domain Validation and Peak Frequency Matching

**Figure 11:**
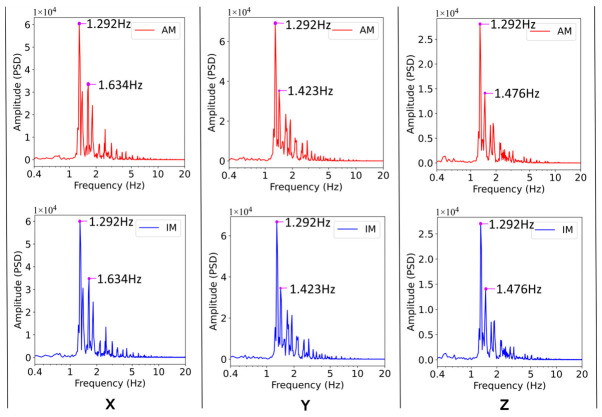
Frequency-domain response comparison between AM (**top row**) and IM (**bottom row**) methods across the X, Y, and Z axes, with the first two peak frequencies labeled, for Test 1.

**Figure 12:**
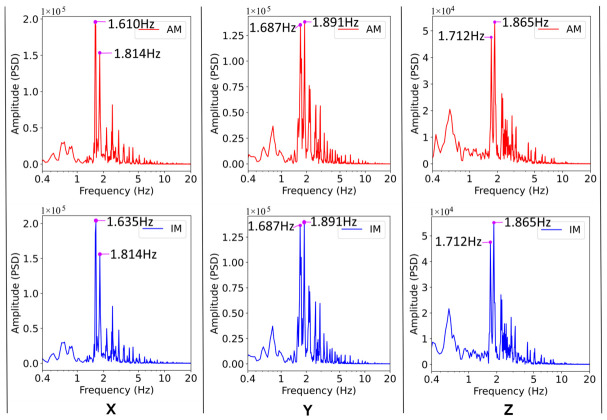
Frequency-domain response comparison between AM (**top row**) and IM (**bottom row**) methods across the X, Y, and Z axes, with the first two peak frequencies labeled, for Test 10.

The frequency-domain performance of the system was evaluated by comparing the first five dominant spectral peaks of the IM with those obtained from AM. Representative comparisons of the best- and worst-performing cases are shown in Figure 11 and Figure 12. Across all 10 tests, 9 showed exact agreement in the first five peak frequencies across all three directions, confirming that the proposed method preserves the dominant dynamic characteristics of the structural response. The one exception occurred in the X-direction (depth axis), where a maximum peak-frequency error of 1.57% was observed, as shown in Figure 12, consistent with stereo reconstruction’s greater sensitivity to out-of-plane motion, where small localization or calibration errors can slightly shift spectral peak estimates.

The reference displacement derived from acceleration using the ω-arithmetic method may introduce amplitude distortion near the low-frequency filter boundary due to the 1/ω^2^ spectral weighting, potential baseline drift from residual sensor offset, and sensitivity to the high-pass cutoff selection. While dominant frequency content is generally well-preserved, displacement amplitudes near the filter bounds may be underestimated or amplified depending on the proximity of structural modes to the cutoff frequency. These limitations are inherent to the integration method rather than to the image-based system under evaluation. The close spectral agreement observed here confirms that both measurement systems capture the same physical vibration content despite differences in modality and signal processing. Upsampling the image-based signals before FFT processing does not artificially improve spectral agreement, as interpolation introduces no new physical frequencies; it provides only a common temporal resolution for comparison.

Across all test conditions, the system achieves 9 out of 10 exact peak-frequency agreement, with a single discrepant case in only one direction showing an error below 2%. These results confirm the framework’s reliability for applications where frequency identification is the primary objective, including modal analysis, resonance tracking, and vibration-based structural health monitoring.

### 3.3. Computational Performance

Processing high-resolution video across hundreds of tracked points must remain feasible within standard post-processing workflows, a non-trivial requirement for image-based structural motion measurement at large scale. Without spatial restrictions on the search region, applying ECC tracking naively to full 4K video produces impractically long processing times. To address this, the present study employs two strategies: reference frame cropping, which confines the search region to a spatially bounded window around each target, and camera-level parallelization, which distributes processing across available CPU threads. Together, these strategies reduce total processing time by up to 178× relative to the naive full-frame baseline, without GPU acceleration or specialized hardware.

Table 4 summarizes the processing times recorded for sequential and camera-level parallel execution across seven test configurations of the large-scale experiment, along with the achieved speedup factors. For the high-density tracking configurations (Tests 1–3, 287–318 tracked points, ~6600 frames per camera), sequential processing required 2062–2150 s, reduced to 1197–1275 s with parallelization, with consistent speedup factors of 1.67–1.72×. Lower-density configurations (Tests 6–7, 64 points) yielded more modest speedups of 1.44–1.56×, reflecting diminishing returns to two-thread parallelism when the per-frame workload is small, and thread overhead constitutes a larger fraction of the total cost. Across all configurations, the speedup remains below the theoretical maximum of 2×, with the gap attributable to shared-memory bus contention and operating-system scheduling latency, which is expected for CPU-bound parallel workloads sharing a common memory hierarchy.

**Figure 13:**
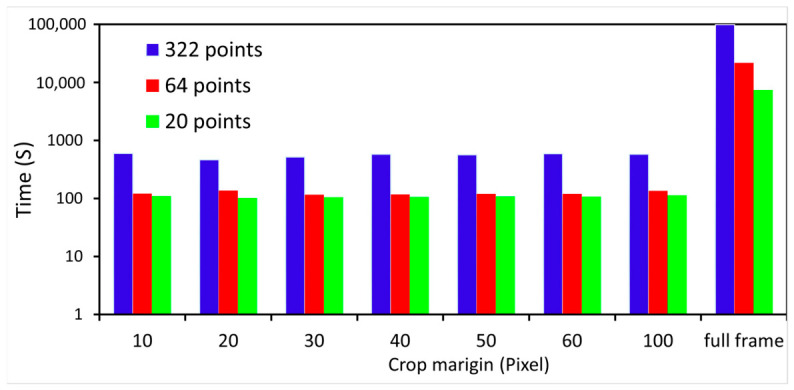
The effect of the cropping margin on tracking time. (Note: vertical axis is in log scale).

The greater efficiency gain arose from restricting the ECC search to a cropped subregion centered on each point’s predicted position. As shown in Figure 13, this modification alone reduced total processing time from approximately 99,066 s (ceiling, full-frame baseline) to 555 s. Combined with camera-level parallelization, the aggregate speedup reaches 178× relative to the naive full-frame baseline. The reductions for the grid and frame surface types were similarly pronounced. Notably, Figure 13 demonstrates that varying the crop margin between 10 and 100 pixels has no practically meaningful effect on processing time across all surface configurations. This plateau confirms that the computational bottleneck in the full-frame case arises from memory-access overhead rather than from the ECC iteration count itself. OpenCV’s ECC implementation operates internally on grayscale representations, producing a grayscale frame buffer of approximately 13 MB (3840 × 3360 × 1 bytes), which exceeds the per-core L2 cache capacity of 1.25 MB of the Intel Core i7-11800H processor used in this study. This case forces repeated high-latency fetches from the shared L3 cache during iterative pixel sampling, substantially increasing per-frame processing cost. Restricting evaluation to a cropped region reduces the active buffer to approximately 40 KB (200 × 200 × 1 bytes), which fits within the 80 KB per-core L1 cache, thus eliminating the memory bottleneck. The dominant source of speedup is therefore the elimination of DRAM access pressure through search-region restriction, rather than a reduction in ECC iteration count.

The combined effect of reference cropping and camera-level parallelism reduces over 27 h of sequential full-frame processing to approximately 10 min. The computational barrier that has limited image-based tracking to low-point-count or short-duration datasets can therefore be effectively removed through these two strategies alone. 

### 3.4. Ceiling System Displacement Analysis

#### 3.4.1. Rigid Frame Motion

**Figure 14:**
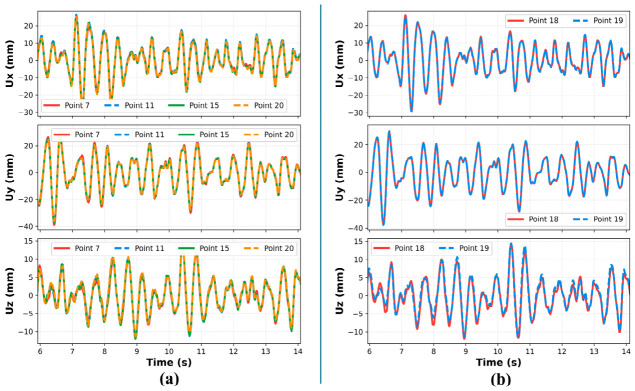
Time-series displacement comparisons in the X, Y, and Z directions to validate synchronous translational motion. (**a**) Relative displacements measured at the four corner joints of the rigid frame; (**b**) relative displacements measured at the shake-table feet.

For a rigid frame, pure translational motion is expected in the absence of rotation. To validate the image-based measurements, two independent comparisons were conducted. First, displacements at the four corner joints of the rigid frame were compared to assess spatial consistency throughout the structure (Figure 14a). Second, displacements at the two adjacent shake-table feet were compared to verify the table’s synchronized operation (Figure 14b). In both cases, peak differences remained below 2 mm in all directions. Additionally, 6-DOF rigid-body motion was evaluated across all ten tests; the estimated rotational components were negligible, confirming that the structural response was dominated by three-dimensional translation.

#### 3.4.2. Ceiling Grid Motion

**Figure 15:**
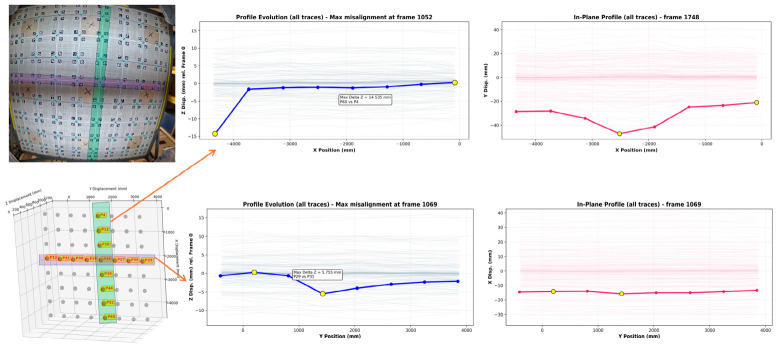
Maximum ceiling grid deformation plots in the vertical direction (X-Z and Y-Z planes) and horizontal direction (X-Y plane); the yellow dots represent the maximum misalignment between ceiling grid nodes, and the thin lines represent the traces of ceiling grid motion.

The three-dimensional displacement response of the selected sample ceiling grid was examined along two orthogonal measurement lines, with tracking performed at grid intersection points over 2339 frames (Figure 15). Along the column direction or orthogonal to the main runners (X-direction, points P4–P60), substantially larger inter-point displacements were recorded than in the row direction. The maximum out-of-plane displacement reached 14.5 mm (Frame 1052, between P60 and P4), and the maximum in-plane displacement reached 26.3 mm (Frame 1748, between P36 and P4). Peak displacements were temporally clustered around Frames 1583 and 1748, with values consistently exceeding 21 mm, indicating a sustained period of pronounced differential displacement concentrated at mid-span nodes.

Along the row direction or parallel to the main runners (Y-direction, points P25–P32), the response was markedly more uniform. The maximum out-of-plane displacement was 5.8 mm (Frame 1069, between P29 and P31), and the maximum in-plane displacement was 5.9 mm (Frame 1737, between P29 and P25). Peak displacements across all projection planes remained below 7 mm throughout.

The consistently larger displacements in the column direction are attributable to a traveling-wave vertical deformation pattern propagating along the grid’s length axis. The restrained and symmetric row-direction response is consistent with a convex in-plane bending mode in which differential displacements between adjacent nodes remain small. Together, these results confirm that the ceiling grid exhibits directionally asymmetric dynamic behavior, with the column axis governing the critical displacement response under the applied loading. This behavior is due to the main runners only running in the row (Y) direction.

#### 3.4.3. Ceiling Panels Motion

**Figure 16:**
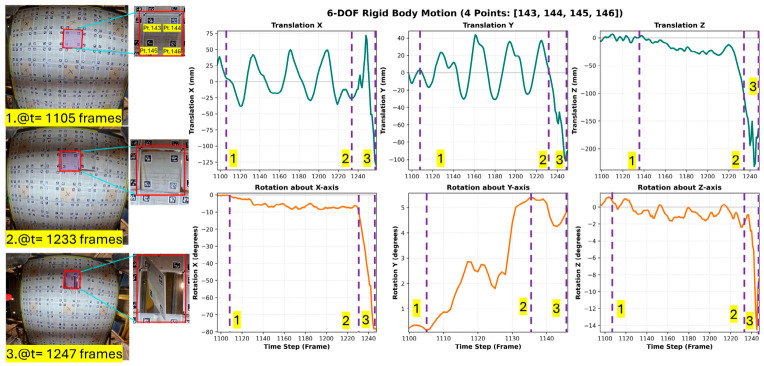
Ceiling panel rigid body motion analysis.

The 6-DOF motion, in terms of translations (Tx, Ty, Tz) and rotations (Rx, Ry, Rz), was reconstructed from the four tracked points to characterize the failure mechanism of ceiling panels. Figure 16 shows the full 6-DOF time-series response within the critical failure window (frames 1105–1247), divided into three distinct phases corresponding to the progressive collapse sequence. The rotational components were derived using the Kabsch algorithm applied to the four tracked points, each providing three independent spatial coordinates, yielding an over-constrained system of 12 observations for 6 unknowns. It is worth noting that coplanar point configurations produce a near-zero singular value in the SVD associated with the out-of-plane direction, rendering in-plane rotations (Rx and Ry) ill-conditioned when the panel remains flat. The implications of this geometric constraint are discussed for each phase below.

In phase 1 (i.e., before frame 1105), the panel undergoes normal translational motion with amplitudes that are consistent with the motion of the ceiling grid, and rotational components remain near zero across all three axes, confirming that the panel behaves as a constrained rigid body with intact grid connections. Although the panel remains approximately planar during this phase, where Rx and Ry are most susceptible to ill-conditioning, the reported near-zero values are consistent with the expected constrained behavior and are not adversely affected by this geometric limitation.

Phase 2 (frames 1105–1233) marks a distinct shift in the panel’s kinematic behavior, triggered by the failure of the grid connection. In-plane translational responses show modest changes as the ceiling grid restraint on the perimeter of the ceiling panel loosens, with the most notable effect in the vertical direction (Tz), where a steady downward displacement reveals a progressive loss of vertical support. At the same time, all three rotational components increase significantly: Rx approaches −10°, Ry reaches approximately +6°, and Rz approximately −2°. This multi-axis rotation is consistent with the panel pivoting about a partially retained edge or corner support following grid disengagement. Critically, the panel’s out-of-plane rotation during this phase breaks the coplanarity condition, thereby improving the conditioning of the Rx and Ry extractions precisely when these components are of greatest interest.

In phase 3 (beyond frame 1233), the panel starts to free-fall following complete detachment from the ceiling grid. Tz displacement drops beyond −200 mm over a short time interval, while Tx and Ty exhibit large erratic fluctuations reflecting unrestrained motion under gravity. Rotational components diverge beyond physically meaningful values, and stereo tracking subsequently fails as the panel exits the calibrated measurement volume. The 6-DOF analysis thereby resolves the complete failure sequence, from constrained vibration through multi-axis rotation to gravitational free fall, providing a level of kinematic detail unattainable with conventional instrumentation.

## 4. Conclusions and Limitations

### 4.1. Conclusions

This study presented a stereo-vision measurement framework for full-field three-dimensional displacement monitoring of suspended ceiling systems subjected to seismic loading on a large-scale shake-table. The framework was designed to address the physical, geometric, and computational constraints of overhead destructive testing environments and validated through a full-scale experiment that continuously tracked hundreds of independent spatial nodes across multiple ceiling panels through seismic excitation sequences. The experimental validation demonstrated RMSE values below 3 mm across all three directions in all test configurations, NRMSE within 2–3% in the majority of tests, and exact peak-frequency agreement between image-derived and accelerometer-derived displacement signals in nine out of ten test cases, confirming that the framework reliably captures both the amplitude and temporal characteristics of the structural dynamic response.

The multi-stage tracking pipeline, combining kinematic warp initialization, localized region-of-interest cropping, histogram equalization, and template-matching fallback, extended reliable trajectory capture significantly beyond the performance envelope of conventional single-step ECC, maintaining tracking fidelity through severe seismic excitations and progressive connection failure up to the limits imposed by the planar template assumption. Reference frame cropping alone reduced per-point, per-camera processing time by up to 89× relative to full-frame ECC tracking; combined with camera-level parallelization, total processing time was reduced from over 27 h to under 10 min without GPU acceleration, making dense multi-point stereo tracking viable within standard post-processing workflows.

The ceiling system displacement analysis revealed directionally asymmetric dynamic behavior, with the vertical-direction measurement line exhibiting substantially larger inter-point displacements, reaching 26.3 mm in-plane and 14.5 mm out-of-plane, compared to the more restrained row-direction response. The 6-DOF rigid-body motion analysis of the failing panel resolved three distinct kinematic phases: constrained oscillatory vibration with near-zero rotation prior to grid failure; progressive multi-axis rotation reaching −75° about the primary axis as grid connections disengaged; and unrestrained gravitational free-fall with vertical displacement exceeding −200 mm following complete panel detachment.

Collectively, these results demonstrate that consumer-grade stereo vision, integrated with purpose-built processing algorithms, provides a practical and scalable alternative to conventional instrumentation for large-scale seismic testing of spatially distributed non-structural systems. The framework establishes a replicable methodology that can be directly adopted in future ceiling-system research and extended to similar non-structural testing scenarios, lowering the barrier to full-field kinematic monitoring in settings where conventional instrumentation is physically or logistically infeasible.

### 4.2. Limitations and Future Work

The accuracy of depth reconstruction is limited by the triangulation geometry at large working distances, where small errors in image-plane feature localization are nonlinearly amplified into depth-coordinate uncertainty, reducing accuracy compared to the in-plane directions. Future deployments should investigate increasing the stereo baseline distance between cameras, adding a third camera to over-constrain the triangulation, or applying photogrammetric bundle adjustment across multiple camera pairs to mitigate this geometric sensitivity.

The stereo synchronization method achieves sub-frame precision but assumes that a sufficient number of spatially distributed common points are simultaneously visible in both camera views during the synchronization window. In scenarios with highly localized failures or severe occlusion, this assumption may be violated, degrading synchronization accuracy.

The 6-DOF rigid-body motion was derived via the Kabsch algorithm using four tracked points. Although translations were validated using accelerometers, direct rotational validation was unavailable. However, the rotational results maintain implicit geometric consistency because they are mathematically extracted from validated spatial coordinates. Future studies should utilize inertial measurement units or motion capture to directly validate these rotational components.

The impact of varying illumination on tracking fidelity remains uncharacterized. Facility conditions such as uneven lighting, glare, or migrating shadows can degrade tracking accuracy and require future evaluation. Furthermore, the applied seismic excitations were limited to peak frequencies below 2.6 Hz. Because higher frequencies proportionally increase inter-frame displacements, systematic evaluation across a broader frequency spectrum is necessary to fully establish the framework’s operational envelope.

The computational results were obtained on a single CPU-based laptop workstation. The framework has not been optimized for GPU acceleration, which could reduce processing times significantly. The current implementation also does not support live streaming analysis, limiting its utility for adaptive experimental control based on real-time displacement feedback.

The present study focuses on a single ceiling typology: a light-gauge steel grid lay-in assembly subjected to tri-directional seismic excitation. The generalizability of the observed failure mechanisms and measurement performance to other configurations, including acoustic tile systems, integrated mechanical service ceilings, and seismically braced assemblies, remains to be established. Extending the framework to a broader range of non-structural components, including partition walls, mechanical equipment, and façade systems, represents a further direction for future research.

## Figures and Tables

**Table 1:** Statistical summary of accuracy assessments for X-axis (Depth direction).

Test	Dominant Frequency (Hz)	RMSE (mm)	NRMSE (%)	MAE (mm)	Peak Error (mm)	Range (mm)
1	1.292	0.391	1.459	0.275	0.243	26.760
2	1.298	0.638	1.224	0.430	0.443	52.068
3	1.294	1.088	1.180	0.704	0.550	92.230
4	1.293	1.909	1.623	1.289	0.264	117.660
5	1.633	1.986	2.149	1.312	7.199	92.402
6	1.628	2.378	2.544	1.550	8.468	93.478
7	1.808	2.548	3.101	1.608	11.216	82.170
8	2.543	1.502	2.679	0.941	0.824	56.078
9	1.635	2.259	2.400	1.538	6.920	94.112
10	1.610	2.483	2.612	1.575	4.481	95.038

**Table 2:** Statistical summary of accuracy assessments for Y-axis (horizontal direction).

Test	Dominant Frequency (Hz)	RMSE (mm)	NRMSE (%)	MAE (mm)	Peak Error (mm)	Range (mm)
1	1.292	0.379	1.473	0.256	0.093	25.700
2	1.298	0.634	1.250	0.413	0.209	50.717
3	1.294	1.100	1.227	0.700	0.158	89.639
4	1.293	2.028	1.739	1.347	0.687	116.590
5	1.422	1.613	1.908	1.112	1.121	84.521
6	1.891	1.848	2.011	1.264	1.154	91.880
7	1.885	1.786	1.948	1.270	3.647	91.669
8	1.888	1.498	2.179	0.934	1.374	68.758
9	1.437	1.972	2.325	1.391	0.370	84.806
10	1.297	1.926	2.117	1.282	0.951	90.980

**Table 3:** Statistical summary of accuracy assessments for Z-axis (vertical direction).

Test	Dominant Frequency (Hz)	RMSE (mm)	NRMSE (%)	MAE (mm)	Peak Error (mm)	Range (mm)
1	1.292	0.217	1.997	0.166	0.122	10.860
2	1.298	0.351	1.618	0.261	0.430	21.682
3	1.294	0.551	1.470	0.390	0.123	37.500
4	1.293	2.869	5.498	1.708	1.119	52.180
5	1.475	0.959	2.173	0.640	2.109	44.113
6	1.864	1.821	3.529	1.098	4.529	51.590
7	1.860	2.970	5.458	1.372	1.822	54.410
8	1.914	0.803	3.124	0.542	0.797	25.692
9	1.466	0.986	2.161	0.664	2.935	45.607
10	1.865	1.987	3.781	1.146	8.055	52.558

**Table 4:** Image tracking time between sequential and camera-level parallel processing.

Test	Tracked Points	Frames Processed (Left/Right)	Sequential Time (s)	Parallel Time (s)	Speedup Factor
1	287	6600/6420	2061.01	1197.59	1.721
2	318	6900/6300	2149.90	1252.85	1.716
3	318	6600/6360	2126.98	1274.30	1.669
4	322	2310/2331	877.82	505.14	1.738
5	323	2340/2340	868.58	506.86	1.714
6	64	2310/2340	182.56	117.12	1.559
7	64	2331/2340	166.41	115.71	1.438

## Data Availability

The source code supporting the findings of this article is openly available on GitHub at https://github.com/Mearge/ParaStereoSync-and-KineViz-3D, version 1.0.0 (accessed on 2 June 2026) under an open-source license. The raw experimental data will be made available by the authors upon reasonable request.

## References

[1] Qi L., Luo Z., Cao Y., Xue J. (2023). Seismic Damage Mechanism and Performance Evaluation of Suspended Ceiling Systems. J. Build. Eng..

[2] Salkhordeh M., Soroushian S. (2026). Seismic performance of suspended ceiling systems; a literature review. J. Earthq. Eng..

[3] Dhakal R.P., MacRae G.A., Hogg K. (2011). Performance of Ceilings in the February 2011 Christchurch Earthquake. Bull. N. Z. Soc. Earthq. Eng..

[4] Lu Y., Mosqueda G., Han Q., Zhao Y. (2018). Shaking table tests examining seismic response of suspended ceilings attached to large-span spatial structures. J. Struct. Eng..

[5] Brandolese S., Fiorin L., Scotta R. (2019). Seismic demand and capacity assessment of suspended ceiling systems. Eng. Struct..

[6] Phan L.T., Taylor A.W. (1996). State of the Art Report on Seismic Design Requirements for Nonstructural Building Components.

[7] Rodgers J., Hassan W., Motter C., Thornley J. (2021). Impacts of the 2018 M7.1 Anchorage Earthquake on Schools. Earthq. Spectra.

[8] Rezvani R., Soroushian S., Zaghi A.E., Maragakis M. (2022). Numerical seismic fragility analysis for suspended ceilings with various geometries. J. Build. Eng..

[9] Feng Y., Li Q., Qu Z. (2023). Frequency-sensitivity of the seismic damage to suspended ceilings. Earthq. Eng. Struct. Dyn..

[10] Filiatrault A., Sullivan T. (2014). Performance-Based Seismic Design of Nonstructural Building Components: The next Frontier of Earthquake Engineering. Earthq. Eng. Eng. Vib..

[11] Gopagani S., Filiatrault A., Aref A.J., Perrone D. (2023). Finite-element modeling for seismic damage estimation of suspended ceiling systems. J. Struct. Eng..

[12] Jun S.-C., Lee C.-H., Bae C.-J., Lee K.-J. (2022). Shake-table seismic performance evaluation of direct- and indirect-hung suspended ceiling systems. J. Earthq. Eng..

[13] Qi L., Kurata M., Ikeda Y., Kunitomo K., Takaoka M. (2021). Seismic evaluation of two-elevation ceiling system by shake table tests. Earthq. Eng. Struct. Dyn..

[14] Magliulo G., Pentangelo V., Maddaloni G., Capozzi V., Petrone C., Lopez P., Talamonti R., Manfredi G. (2012). Shake table tests for seismic assessment of suspended continuous ceilings. Bull. Earthq. Eng..

[15] Jiang H., Wang Y., Huang Y. (2022). Shaking table tests and numerical modeling of discontinuous suspended ceiling system with free boundary condition. Eng. Struct..

[16] Fiorino L., Bucciero B., Landolfo R. (2019). Evaluation of seismic dynamic behaviour of drywall partitions, façades and ceilings through shake table testing. Eng. Struct..

[17] Kim S.-Y., Lee S.-J., Choi K.-K. (2025). Dynamic characteristics of multi-degree-of-freedom frame systems derived from vision-based displacement measurement using consumer-grade camera video. J. Civ. Struct. Health Monit..

[18] Qin W., Wang F., Hu S., Shimasaki K., Ishii I. (2025). Multi-camera 3d digital image correlation with pointwise-optimized model-based stereo pairing. Sensors.

[19] Yang R., Li Y., Zeng D., Guo P. (2022). Deep DIC: Deep Learning-based digital image correlation for end-to-end displacement and strain measurement. J. Mater. Process. Technol..

[20] Shao Y., Li L., Li J., An S., Hao H. (2021). Computer vision based target-free 3D vibration displacement measurement of structures. Eng. Struct..

[21] Ji X., Miao Z., Kromanis R. (2020). Vision-based measurements of deformations and cracks for RC structure tests. Eng. Struct..

[22] Yang Y.-S. (2019). Measurement of dynamic responses from large structural tests by analyzing non-synchronized videos. Sensors.

[23] Marendić A., Gajski D., Duvnjak I., Kosor A. (2026). Experimental accuracy evaluation of UAV-based homography for static and dynamic displacement monitoring of structures. Sensors.

[24] Heo J., Cho H.-C., Hwang J.-H., Ju H. (2026). Image-based analysis of the flexural behavior of reinforced concrete beams. Eng. Struct. Civ. Eng..

[25] Yang Y.-S., Xue Q., Chen P.-Y., Weng J.-H., Li C.-H., Liu C.-C., Chen J.-S., Chen C.-T. (2020). Image analysis applications for building inter-story drift monitoring. Appl. Sci..

[26] Gioiella L., Micozzi F., McBain M., Morici M., Zona A., Dall’Asta A., Simpson B.G., Barbosa A.R. (2025). Vision-based monitoring of absolute and relative displacements in multistory buildings during full-scale shake-table tests. Struct. Control Health Monit..

[27] He J., Wang Y., Xu Y., Xu X. (2025). development of a vision-based dynamic response measurement method using novel targets in a large-scale shaking table test. Structures.

[28] Yang Y.-S., Wu C., Hsu T.T.C., Yang H.-C., Lu H.-J., Chang C.-C. (2018). Image analysis method for crack distribution and width estimation for reinforced concrete structures. Autom. Constr..

[29] Wu T., Tang L., Shao S., Zhang X., Liu Y., Zhou Z., Qi X. (2022). Accurate structural displacement monitoring by data fusion of a consumer-grade camera and accelerometers. Eng. Struct..

[30] Yang Y.-S., Chang C.-H., Wu C. (2019). Damage indexing method for shear critical tubular reinforced concrete structures based on crack image analysis. Sensors.

[31] Kuddus M.A., Li J., Hao H., Li C., Bi K. (2019). Target-free vision-based technique for vibration measurements of structures subjected to out-of-plane movements. Eng. Struct..

[32] Lin S.-H., Chen E.-T., Xiao J.-J., Chang C.-Y. (2024). Stereo digital image correlation (3D-DIC) for non-contact measurement and refinement of delta robot arm displacement and jerk. Int. J. Adv. Manuf. Technol..

[33] Seyfu M.K., Yang Y.-S. (2025). A Stereo synchronization method for consumer-grade video cameras to measure multi-target 3D displacement using image processing in shake table experiments. Sensors.

[34] Du W., Lei D., Zhu F., Bai P., Zhang J. (2024). A non-contact displacement measurement system based on a portable smartphone with digital image methods. Struct. Infrastruct. Eng..

[35] Guo Q., Gao W., Yang T.Y., Lu X. (2025). Hierarchical nondestructive detection of full-scene suspended ceiling systems using point cloud. Comput.-Aided Civ. Infrastruct. Eng..

[36] Wang P., Xiao J., Kawaguchi K., Wang L. (2022). Automatic ceiling damage detection in large-span structures based on computer vision and deep learning. Sustainability.

[37] Han Q., Yan S., Wang L., Kawaguchi K. (2023). Ceiling damage detection and safety assessment in large public buildings using semantic segmentation. J. Build. Eng..

[38] Evangelidis G.D., Psarakis E.Z. (2008). Parametric image alignment using enhanced correlation coefficient maximization. IEEE Trans. Pattern Anal. Mach. Intell..

[39] OpenCV OpenCV Modules. https://docs.opencv.org/4.x/.

[40] Flude C.C., Lau D.T., Erochko J.A. Experimental Seismic Testing of Suspended Ceilings Using a Six Degree-of-Freedom Shake Table System. Proceedings of the Experimental Mechanics in Engineering and Biomechanics-Proceedings ICEM20: 20th International Conference on Experimental Mechanics.

[41] Garrido-Jurado S., Muñoz-Salinas R., Madrid-Cuevas F.J., Marín-Jiménez M.J. (2014). Automatic Generation and Detection of Highly Reliable Fiducial Markers under Occlusion. Pattern Recognit..

[42] Guo K., Ye H., Gu J., Chen H. (2021). A novel method for intrinsic and extrinsic parameters estimation by solving perspective-three-point problem with known camera position. Appl. Sci..

[43] Raza S.N., Rehman H.R., Lee S.G., Choi G.S. Artificial Intelligence based Camera Calibration. Proceedings of the 15th International Wireless Communications & Mobile Computing Conference (IWCMC).

[44] Kaiser M., Brusa T., Bertsch M., Wyss M., Ćuković S., Meixner G., Koch V.M. (2024). Extrinsic calibration for a modular 3D scanning quality validation platform with a 3D checkerboard. Sensors.

[45] Jing X., Han F., Ding X., Wang Y., Xiong R. Intrinsic and Extrinsic Calibration of Roadside Lidar and Camera. Proceedings of the 2022 China Automation Congress (CAC).

[46] Otsu N. (1975). A threshold selection method from gray-level histograms. Automatica.

[47] Meyer J., Giraud F., Wüthrich J., Pollefeys M., Fürnstahl P., Calvet L. (2026). RocSync: Millisecond-accurate temporal synchronization for heterogeneous camera systems. Sensors.

[48] Kabsch W. (1976). A solution for the best rotation to relate two sets of vectors. Found. Crystallogr..

